# Differences in Cell Morphometry, Cell Wall Topography and Gp70 Expression Correlate with the Virulence of *Sporothrix brasiliensis* Clinical Isolates

**DOI:** 10.1371/journal.pone.0075656

**Published:** 2013-10-07

**Authors:** Rafaela A. Castro, Paula H. Kubitschek-Barreira, Pedro A. C. Teixeira, Glenda F. Sanches, Marcus M. Teixeira, Leonardo P. Quintella, Sandro R. Almeida, Rosane O. Costa, Zoilo P. Camargo, Maria S. S. Felipe, Wanderley de Souza, Leila M. Lopes-Bezerra

**Affiliations:** 1 Laboratório de Micologia Celular e Proteômica, Instituto de Biologia Roberto Alcantara Gomes, Universidade do Estado do Rio de Janeiro UERJ, Rio de Janeiro, Brazil; 2 Laboratório de Biologia Molecular, Instituto de Ciências Biológicas, Universidade de Brasília, Brasília, Brazil; 3 Departamento de Patologia e Laboratórios, Faculdade de Ciências Médicas, UERJ, Rio de Janeiro, Brazil; 4 Departamento de Análises Clínicas e Toxicológicas, Faculdade de Ciências Farmacêuticas, Universidade de São Paulo, São Paulo, Brazil; 5 Laboratório de Micologia, Hospital Universitário Pedro Ernesto, UERJ, Rio de Janeiro, Brazil; 6 Departamento de Microbiologia, Imunologia e Parasitologia, Universidade Federal de São Paulo, São Paulo, Brazil; 7 Laboratório de Ultraestrutura Celular Hertha Meyer, Universidade Federal do Rio de Janeiro, Rio de Janeiro, Brazil; The University of Texas at San Antonio, United States of America

## Abstract

Sporotrichosis is a chronic infectious disease affecting both humans and animals. For many years, this subcutaneous mycosis had been attributed to a single etiological agent; however, it is now known that this taxon consists of a complex of at least four pathogenic species, including *Sporothrix schenckii* and *Sporothrix brasiliensis*. Gp70 was previously shown to be an important antigen and adhesin expressed on the fungal cell surface and may have a key role in immunomodulation and host response. The aim of this work was to study the virulence, morphometry, cell surface topology and gp70 expression of clinical isolates of *S. brasiliensis* compared with two reference strains of *S. schenckii*. Several clinical isolates related to severe human cases or associated with the Brazilian zoonotic outbreak of sporotrichosis were genotyped and clustered as *S. brasiliensis*. Interestingly, in a murine subcutaneous model of sporotrichosis, these isolates showed a higher virulence profile compared with *S. schenckii*. A single *S. brasiliensis* isolate from an HIV-positive patient not only showed lower virulence but also presented differences in cell morphometry, cell wall topography and abundant gp70 expression compared with the virulent isolates. In contrast, the highly virulent *S. brasiliensis* isolates showed reduced levels of cell wall gp70. These observations were confirmed by the topographical location of the gp70 antigen using immunoelectromicroscopy in both species. In addition, the gp70 molecule was sequenced and identified using mass spectrometry, and the sequenced peptides were aligned into predicted proteins using Blastp with the *S. schenckii* and *S. brasiliensis* genomes*.*

## Introduction

Sporotrichosis is the most frequent subcutaneous mycosis in Latin America [1-3]. Until recently, this disease had always been attributed to a single etiological agent, the dimorphic fungus *Sporothrix schenckii* [4,5]. A recent phylogenetic study proposed that *S. schenckii* is actually a complex of cryptic species, at least five of which have clinical interest: *S. globosa*, *S.* brasiliensis, *S.* luriei, *S.* mexicana and *S. schenckii* *sensu stricto* [6-8]. However, when several isolates were genotyped more recently using different markers, it was found that *S. mexicana* seems to cluster with environmental species in the *Sporothrix pallida* complex, and these species were proposed to be accidentally pathogenic [9]. This hypothesis was supported by thermotolerance evaluation of the isolates analyzed compared with clinical isolates of the *S. schenckii* complex [10]. Phenotypic and genotypic features of different isolates within this complex were associated with their geographical distribution, virulence capacity or clinical manifestation of sporotrichosis [11-14]. In addition, several reports have shown that more than one species can be isolated within the same endemic area [1,15-17]. Two pathogenic species have been associated with endemic areas in Brazil, *S. schenckii* and *S. brasiliensis* [10,15].

A large variety of clinical manifestations of sporotrichosis have been described, but there is no clear explanation for the incidence of severe and disseminated forms of sporotrichosis in immunologically healthy patients [18]. In addition to the presence of more than one pathogenic species within the *S. schenckii* complex, other biological factors inherent to the pathogen may be relevant to the outcome of an infectious disease, as proposed for other pathogens [19]. Therefore, the multifactorial nature of the virulence of pathogenic microorganisms should be considered. Recently, it was shown that zoonotic outbreaks registered in Brazil are caused mainly by *S. brasiliensis*, which is recognizably more thermotolerant than the other species of the *Sporothrix* complex. However, other relevant factors for the pathogenesis of these organisms need to be elucidated, as the mechanisms involving the colonization and rapid aggravation of the disease are still not clearly understood. Other virulence factors already known in *S. schenckii* are extracellular enzymes [20-22], adhesins [23,24] and melanin [25,26]. The major adhesin described on the cell surface of *S. schenckii*, gp70, has been shown to mediate the binding of yeast cells to the dermal matrix and to fibronectin [24,27]. Gp70 also plays a pivotal role as an antigen, which can modulate the host immune response [28].

Little is known about the virulence factors of isolated cryptic species of the *Sporothrix schenckii* complex. In this context, the emergence and pathogenicity of *S. brasiliensis* leads to the necessity to elucidate the virulence mechanisms of this new species [14,29].

The aim of the present work is to study the virulence, morphometry, cell surface ultrastructure and gp70 cell wall expression of *S. brasiliensis* clinical isolates compared with *S. schenckii* reference strains. In addition, the gp70 peptide sequence was revealed based on the genome database of both species.

## Materials and Methods

### Ethics statement

All procedures were performed in strict accordance with Brazilian Federal Law 11,794 for Procedures of the Scientific Use of Animals and with the National Health Animal Care Guidelines. The animal experiments were performed to minimize suffering of the animals and were approved by the Institutional Ethics Committee of Instituto de Biologia Roberto Alcantara Gomes, Universidade do Estado, Rio de Janeiro, Brazil (CEA-IBRAG-UERJ, process number CEA/027/2010).

### Microorganism and culture conditions

All isolates used throughout this study are listed in Table 1, including the geographical, genotype and clinical origin when pertinent. The yeast phase of each strain was obtained by cultivation in Brain Heart Infusion broth (Oxoid, Hampshire, UK) at 37 °C for 7 days, as previously described [30]. For *in vivo* experiments, the mycelia phase of each strain was grown in Sabouraud broth (Difco, Detroit, USA) for 5 days at room temperature, and the conidia were isolated from hyphae using a Buchner funnel and gauze (see Figure S2). For the scanning electron microscopy (SEM) experiments, the mycelial phase of *S. schenckii* isolates 1099-18 and IPEC 15383 and *S. brasiliensis* isolates IPEC 17943 and 5110 were cultivated in Sabouraud-agar discs placed between coverslips and incubated at room temperature for 15 days.

**Table 1:** Clinical information and genotype of *Sporothrix* spp. isolates used in the study.

**Isolate**	**Host / Clinical Form**	**Geographical Origin (State, Country)**	**Genotype**	***GenBank* Accession Number for** *cal * ***locus***
1099-18	Human / LC^a^	USA	*S. schenckii*	JF313360 –this study
IPEC 15383	Human / LC and EC^a^ (osteoarticular)	Rio de Janeiro, Brazil	*S. schenckii*	JF313361 - this study
IPEC 17943	HIV+ Human/FC^a^ and meningitis	Rio de Janeiro, Brazil	*S. brasiliensis*	JF313362 - this study
5110	Cat / Cutaneous	Rio de Janeiro, Brazil	*S. brasiliensis*	JF313351 - this study
Ss54 (CBS132990)	Cat / ND^b^	Rio Grande do Sul, Brazil	*S. brasiliensis*	Rodrigues et al., 2013
HUPE 114500	Human / Meningeal	Rio de Janeiro, Brazil	*S. brasiliensis*	KF048980 - this study
HUPE 114158	Human / cutaneous with facial destruction	Rio de Janeiro, Brazil	*S. brasiliensis*	KF048981 - this study
UFTM01	Human /CD^a^ and EC (endocarditis)	Minas Gerais, Brazil	*S. brasiliensis*	Silva-Vergara et al., 2012

a-LC_Lymphocutaneous sporotrichosis; EC_Extracutaneous; FC_Fixed cutaneous; CD_cutaneous disseminated.b-ND - Not described.

### Genotyping of clinical isolates of the *Sporothrix* genus

About 500 mg of each isolate in mycelium form was macerated in liquid nitrogen using pestle and mortle. DNA extractions were performed using the DNAeasy Plant Mini Kit (Qiagen, Venlo, NLD). DNA integrity was checked by electrophoresis in 0.8% agarose gel stained with 0,5µg/ml of ethidium bromide and samples were quantified in NanoDROP 2000c (Thermo). Primers were designed for amplification of the calmodulin (cal) gene (forward 5' GARTWCAAGGAGGCCTTCTC 3' and reverse 5' TTTTGCATCATGAGTTGGAC) for species delineation [6]. PCR reactions were carried out using 25 ng of genomic DNA from each of 7 isolates. The sequences of the amplicons were deduced by automatic capillary Sanger sequencing in a MegaBACE^TM^ 500 machine using the DYEnamic^TM^ ET Dye Terminator Kit (GE Healthcare, Piscataway, NJ, USA). The fragments were sequenced on both strands to increase the quality of the sequence data (*phred*>30). To assess the phylogenetic distribution of the isolates, sequences of the cal locus from type strains of *S. schenckii* (AM117437), *S. brasiliensis* (AM116899) and *S. globosa* (AM116908) were accessed from GENBANK and added to the analysis. The sequences were compared with the NCBI nucleotide databank using the Blastn tool. Sequence alignments were performed using the ClustalW algorithm [31] implemented in the BioEdit software [32]. The retrieved alignments were manually edited to avoid mispaired bases. Phylogenetic analysis was conducted in MEGA5 [33]. The phylogenies were inferred using the Maximum Likelihood and Neighbor-Joining methods, and bootstrap searches were performed to check branch fidelity.

### Experimental infection

Groups of 10 male BALB/c mice (2 months old and weighing approximately 30 g) were maintained in temperature-controlled rooms with *ad libitum* access to food and water throughout the experiments. Each mouse was subcutaneously inoculated in the dorsal sacral region with 10^7^ conidia suspended in sterile PBS (Figure S2). The uninfected control group was injected with sterile PBS. To confirm the cell count of each inoculum, an appropriate sample of the conidia cell suspension was plated onto BHI (Brain Heart Infusion) agar plates; after 7 days of incubation, the number of colonies was determined. Infected and control mice were monitored daily for at least 40 days to evaluate the progression of the disease. The parameters determined were the extension of the primary skin lesion and the CFU. Three mice of each group were euthanized 40 days post-infection, and the skin lesion, lungs and spleen were removed to assess the fungal load, to evaluate dissemination to internal organs and to perform histopathological analysis, as detailed below.

### Histopathology

Skin lesions specimens were collected, fixed in 10% buffered formalin, processed, embedded in paraffin and sectioned in a microtome. Slides were analyzed by one pathologist, blind to the fungus species and strain. Fungal burden was analyzed in Gomori-Grocott methenamine-silver (GMS) stained slides. Assessment of tissue reaction was performed on hematoxylin and eosin (HE)-stained slides and included observation of the intensity and organization of neutrophilic and phagocytic mononuclear infiltrates in semi-quantitative scales. Fungal localization in tissue was observed in PAS-stained slides, and the expected extracellular locations were in abscesses, in necrotic tissue and in normal tissue. Furthermore, intracellular fungal structures were also searched for, especially in phagocytic mononuclear cells.

### Preparation of cell wall extracts

Cell wall protein extraction was performed as previously described [34]. Briefly, yeast cells were collected and washed with ice-cold 25 mM Tris/HCl buffer (pH 8.5), likewise used in the subsequent extraction step. Cells were further incubated with an extraction solution (2 mM DTT, 40 µM leupeptin, 1mM PMSF and 5 mM EDTA in 25 mM Tris/HCl, pH 8.5) at 4 °C for 2 h with mild agitation. The supernatant containing the cell surface proteins was collected and concentrated. The proteins were then precipitated with trichloroacetic acid/acetone as previously described [35] and quantified by Bradford assay (Bio-Rad, Hercules, CA, USA).

### Western blot

A monoclonal antibody raised against the gp70 antigen [28], mAb P6E7, was used to evaluate the gp70 expression on the surface of *S. schenckii* and *S. brasiliensis*.

Samples of 5 µg of the cell wall extracts from two reference strains of *S. schenckii* and six *S. brasiliensis* isolates (Table 1) were separated by electrophoresis in a 12% SDS-polyacrylamide gel [36]. Subsequently, proteins were electrotransferred to Hybond ECL nitrocellulose membranes (GE Healthcare) at 50 V for 120 min in 20% methanol, 25 mM Tris, 96 mM glycine (pH 8.3) in a Mini-Protean II Cell (Bio-Rad, USA). Next, the membranes were blocked with 5% skim milk in TBS-T (50 mM Tris, 0.15 M NaCl, 0.1% Tween 20), washed in 1% skim milk in TBS-T and incubated with mAb P6E7 1:1000 for 2 h at room temperature under constant agitation. The membrane was washed in 1% skim milk in TBS-T and incubated with horseradish peroxidase (HRP)-conjugated anti-mouse IgG antibodies diluted 1:4000 according to manufacturer instructions (Kirkegaard & Perry Laboratories, Gaithersburg, MD, USA). After a washing step, the membrane was developed using an ECL Plus kit (GE Healthcare), and the images were obtained using a Typhoon Trio scanner (GE Healthcare).

### Proteomic identification of the 70 kDa cell surface component

For the identification of gp70, cell wall extracts of *S. schenckii* and *S. brasiliensis* and a highly purified gp70 antigen from strain M-64 were used. Protein bands recognized by mAb P6E7 were manually excised from Coomassie-stained SDS-PAGE 1D gels. The gel pieces were destained with ultrapure water overnight, washed twice with 25mM ammonium bicarbonate/50% acetonitrile (ACN) for 30 min, shrunk with 100% ACN for 10 min and vacuum-dried. The gel pieces were then incubated with 12.5 ng/µl sequencing-grade trypsin (Promega, Madison, WI, USA) in 25 mM ammonium bicarbonate overnight at 37°C. After digestion, the supernatants were separated. Peptides were extracted from the gel pieces first into 0.5% trifluoroacetic acid/50% ACN twice for 1 h and then into 100% ACN once for 20 min. All extracts were pooled, and the volume was reduced using a SpeedVac. The cell wall extraction and identification protocols were previously described by Kubitschek-Barreira et al. [34]

After digestion, the supernatant was collected, and 0.5 µl was spotted on a MALDI target plate (Applied Biosystems, Foster City, CA, USA), mixed subsequently with 0.5 µl of matrix (3 mg/ml of α-cyano-4-hydroxy-trans-cinnamic acid; Sigma, St. Louis, MO, USA) diluted in 0.1% trifluoroacetic acid-acetonitrile/H _2_O (1:1, v/v) and allowed to air dry for 5 min at room temperature. The samples were analyzed using a 5800 AB Sciex ToF/ToF 5800 mass spectrometer (Applied Biosystems) in manual mode. Initially, a MALDI MS spectrum was acquired from each spot (700 shots/spectrum), and the ten peaks with a major signal-to-noise ratio in each spectrum were manually selected for MS/MS analysis (4,000 shots/spectrum). All mass spectra were externally calibrated using the 4700 Proteomics Analyzer Mass Standards Kit (Applied Biosystems). The proteins were identified using the Protein Pilot software to perform a search against the *S. schenckii* and *S. brasiliensis* genome database, constructed with curated genome information (unpublished data). A False Discovery Rate (FDR) of 1% was applied to ensure the quality of the data. The gp70 sequences of *S. schenckii* and *S. brasiliensis* were aligned to observe possible differences in the protein sequences between these two species using the NCBI Blastp tool. The identified sequences of *S. schenckii* and *S. brasiliensis* gp70 were deposited at EMBL-EBI.

Moreover, the sequences of gp70 were analyzed using several predictors (EnsembleGly, NetPhos 2.0, SignalIP 4.1 and BigPI) to search for glycosylation and phosphorylation sites and for a secretion signal and GPI anchor. To evaluate the phylogenetic distribution in other Sordariomycetes, the predicted amino acid sequences of *S. schenckii* and *S. brasiliensis* were compared with the NCBI protein database using Blastp, and homologues from other Sordariomycete fungi were used for phylogenetic analysis. Retrieved sequences were aligned and manually edited to avoid mispaired bases. Phylogenetic analyses were conducted in MEGA5 [33] using the Maximum Likelihood method, and the best amino acid evolutive model was selected under AIC (Akaike Information Criterion). Bootstrap searches were performed to check branch fidelity and were added next to the branches. Functional analysis of the gp70 protein sequence was performed using IterPro (http://www.ebi.ac.uk/interpro/), and domains and important sites were annotated.

### Scanning electron microscopy

The yeast and/or the mycelial phases of the reference isolates of *S. schenckii* (1099-18, IPEC 15383) and clinical isolates of *S. brasiliensis* (IPEC 17943 and 5110) were fixed with a solution containing 2.5% glutaraldehyde, 4% freshly prepared formaldehyde in 0.1 M cacodylate buffer (pH 7.2) for 1 h at room temperature and then adhered on coverslips placed in 24-well plates with poly-L-lysine. The coverslips were washed with cacodylate buffer and postfixed with 1% OsO_4_, 0.8% K_4_Fe(CN)_6_·3H_2_O for 1 h. Next, the coverslips were washed, dehydrated with ethanol, critical-point dried with CO_2_ and coated with gold. Observations were performed on a field emission Jeol JSM6340-F or FEI Quanta 250 scanning electron microscope. The yeast and conidial area was measured using the ImageJ software (National Institutes of Health, Bethesda, MD USA).

For the interaction assays of the isolates with mAb P6E7, the yeast cells were fixed with a solution containing 0.1% glutaraldehyde, 1% formaldehyde in 0.1 M cacodylate buffer (pH 7.2) for 1 h at room temperature. After washing, the cells were blocked with NH_4_Cl in cacodylate buffer for 30 min at room temperature. The cells were washed and then adhered on coverslips placed in 24-well plates with poly-L-lysine. The coverslips were incubated with mAb P6E7 (100 µg ml^-1^) for 1 h at 37 °C. The cells were then washed and incubated with 15 nm colloidal gold-labeled anti-mouse IgG diluted 1:50 in PBS for 1 h at 37 °C. The yeasts were postfixed, dehydrated, dried and coated with carbon. Observations were made on a field emission Jeol JSM6340-F scanning electron microscope fitted with a backscatter electron detector.

### Transmission electron microscopy

Yeast cells of *S. schenckii* and *S. brasiliensis* clinical isolates were fixed with a solution containing 0.1% glutaraldehyde, 1% freshly prepared formaldehyde in 0.1 M cacodylate buffer(pH 7.2) for 1 h at room temperature. After washing, the cells were blocked with NH_4_Cl in cacodylate buffer for 30 min at room temperature. Samples were then washed in cacodylate buffer, dehydrated in a graded series of ethanol and embedded in Unicryl resin. Ultrathin sections were incubated with mAb P6E7 (25 µg ml^-1^) in PBS for 1 h, at 37 °C. After washing, the sections were incubated with 10 nm colloidal gold-labeled anti-mouse IgG diluted 1:100 in PBS for 1 h at 37 °C and then stained with aqueous uranyl acetate and alkaline lead citrate and examined in a ZEISS 100 Transmission Electron Microscope.

### Statistical analysis

Statistical analysis was performed by variance analysis (ANOVA) followed by Tukey’s post-test to compare the average lesion diameters among the experimental groups. For the other data, statistical analysis was assessed by analysis of variance followed by Student’s *t*-test. In all cases, *P*<0.05 was considered statistically significant.

## Results

### Phylogenetic analysis of the *Sporothrix* isolates

The phylogenetic distribution of the isolates analyzed places them into *S. schenckii* and *S. brasiliensis* species. The isolates HUPE 114158, HUPE 114500, 5110, Ss54 and IPEC 17943 clustered together with the *S. brasiliensis* type strain IPEC 16490T with high bootstrap values, sharing 99-100% sequence identity. Isolates IPEC 15383 and 1099-18 clustered with the *S. schenckii* type strain CBS 359.36T, supported by high bootstrap values, sharing 99-100% sequence identity (Supplementary material - Figure S1). The strain details such as host, origin and Genbank accession number are shown in Table 1.

### Virulence of *S. brasiliensis* clinical isolates in a subcutaneous murine model

**Figure 1:**
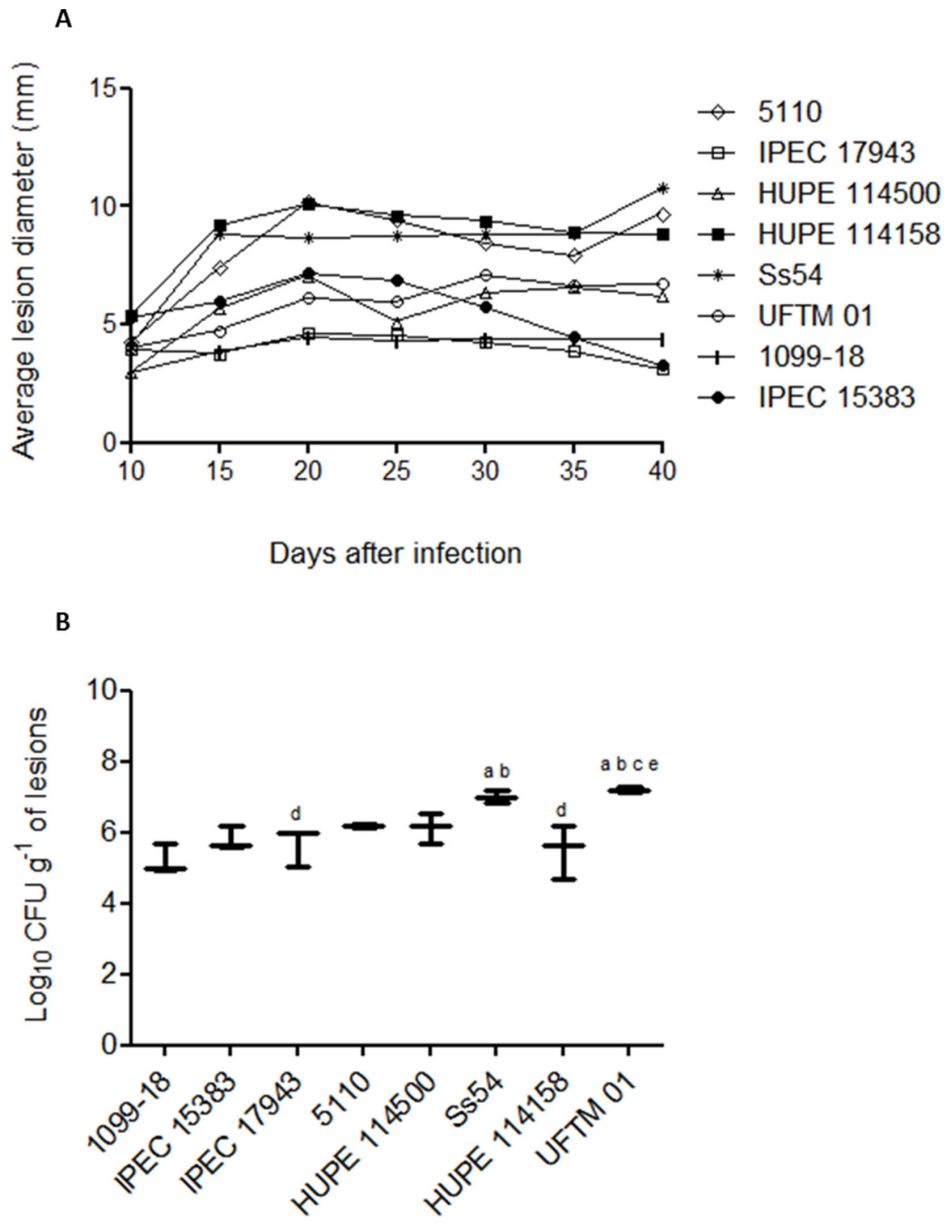
Virulence profile in a murine model of cutaneous sporotrichosis. BALB/c mice were subcutaneously infected with 10^7^ conidia of clinical isolates of *S. schenckii* or *S. brasiliensis*. (**A**) Evolution of primary lesions in the dorsal sacral region of mice infected with *S. brasiliensis* and *S. schenckii* isolates. (**B**) Fungal load of the primary cutaneous lesions determined 40 days post-infection. ^a^ p<0.05 relative to 1099-18 isolate; ^**b**^ p<0.05 relative to IPEC 15383 isolate; ^**c**^ p<0.05 relative to IPEC 17943 isolate; ^**d**^p<0.05 relative to Ss54 isolate; ^**e**^ p<0.05 relative to HUPE 114158 isolate.

To evaluate the pathogenicity of the six clinical isolates of *S. brasiliensis* and to compare them with two reference strains of *S. schenckii* (1099-18, IPEC15383), as detailed in Table 1, a subcutaneous murine model of sporotrichosis was established as illustrated in the supplementary material (Figure S2). Male BALB/c mice were subcutaneously inoculated with 10^7^ conidia of each strain, and the progression of the disease was followed up to 40 days post-infection. The progression of subcutaneous sporotrichosis was determined by the evolution of the primary skin lesion (Figure 1A) and the development of secondary lesions (Table 2). The fungal burden in the primary subcutaneous lesion was determined by CFU counts followed by histopathology (Figure 1B, Figures S3 and S4). Additionally, possible dissemination to the internal organs (spleen and lungs) of the infected animals was investigated (Table 2). In general, compared with the *S. schenckii* reference strains, the *S. brasiliensis* clinical isolates exhibited high pathogenicity with a single exception, IPEC 17943 isolate (Figure 1). The *S. brasiliensis* clinical isolates Ss54, 5110 and HUPE 114158 were shown to induce progressive and/or persistent skin lesions in mice and a higher capacity to disseminate (Figure 1A and Table 2) and were thus considered hypervirulent. The *S. brasiliensis* UFTM 01 and HUPE 114500 isolates presented mild virulence. A single *S. brasiliensis* isolate, IPEC 17943, showed a lower virulence profile and behaved similarly to the *S. schenckii* reference strains (Figure 1A and Table 2). Independently of the virulence profile, it was possible to recover the fungus from the primary skin lesion 40 days post-infection, although a higher fungal burden was observed for the more virulent strains (Figure 1B). Interestingly, for all mice infected with the lower virulence strains, the appearance of secondary lesions was not observed. In contrast, the 5110 isolate of *S. brasiliensis* showed a high dissemination capacity with a significant fungal load observed in the lungs and spleen, leading to 100% mortality of infected mice within 60 days post-infection (Table 2).

**Table 2:** Evaluation of the progression of sporotrichosis caused by *S. brasiliensis* and *S. schenckii* in a subcutaneous murine model.

**Strain**	**Dissemination^a^,^b^**		**Secondary lesions**	**Death^c^**	**Regression of the primary lesion**	**Observations**
	**Lung**	**Spleen**				
**Ss54**	++	++	1/5	1/5	2/5	Secondary open lesions along the tail; macroscopic lesions in the liver.
**5110**	++	++	0/5	5/5	2/5	Inflammation in the paw and posterior leg joints (arthritis); macroscopic lesions in the liver; all mice died within 60 days post-infection.
**HUPE 114158**	++	++	2/4	1/4	0/4	Persistent cutaneous lesions (more than 80 days)
**UFTM 01**	+	+/-	3/5	1/5	1/5	Persistent cutaneous lesions (more than 80 days); inflammation in the paw and joints (arthritis); macroscopic lesions in the liver.
**HUPE 114500**	+/-	+	1/5	0/5	0/5	Persistent cutaneous lesions (more than 80 days)
**IPEC 17943**	+/-	+/-	0/5	0/5	4/5	−
**1099-18**	+	+	0/5	0/5	4/5	−
**IPEC 15383**	+/-	+/-	0/5	0/5	4/5	Secondary ulcerated lesions along the tail; inflammation in the posterior leg joints.

a Dissemination was determined by CFU in lung and spleen specimens collected 40 days post-infection.

b Relative CFU counts (+/-) 20 colonies; (+) 20 to 120 colonies and (++) >120 colonies per Petri dish (the tissue specimens were ressuspended in the same proportion g tissue/mL).

c Mortality up to 60 days post-infection.

Histopathological analysis indicates that the *S. brasiliensis* strains showed the formation of few or no granulomes in the infected tissues with the exception of the lower virulence IPEC17943 strain. In this specific case, the granulome formation in skin lesions was similar to the aspect observed for *S. schenckii* (supplementary material - Figure S4). Interestingly, only *S. schenckii* yeasts were found inside phagocytes (data not shown).

### Morphological comparative analysis of *S. schenckii* and *S. brasiliensis*

For the morphometric and ultrastructural studies, two isolates of each species were selected. In the specific case of *S. brasiliensis*, the isolates showing the lower and higher virulence profiles were chosen. The *S. schenckii* 1099-18 and IPEC 15383 strains were considered reference model strains, as they have been extensively studied previously [4,24]. Both strains were already deposited in the American Type Culture Collection (ATCC).

**Figure 2:**
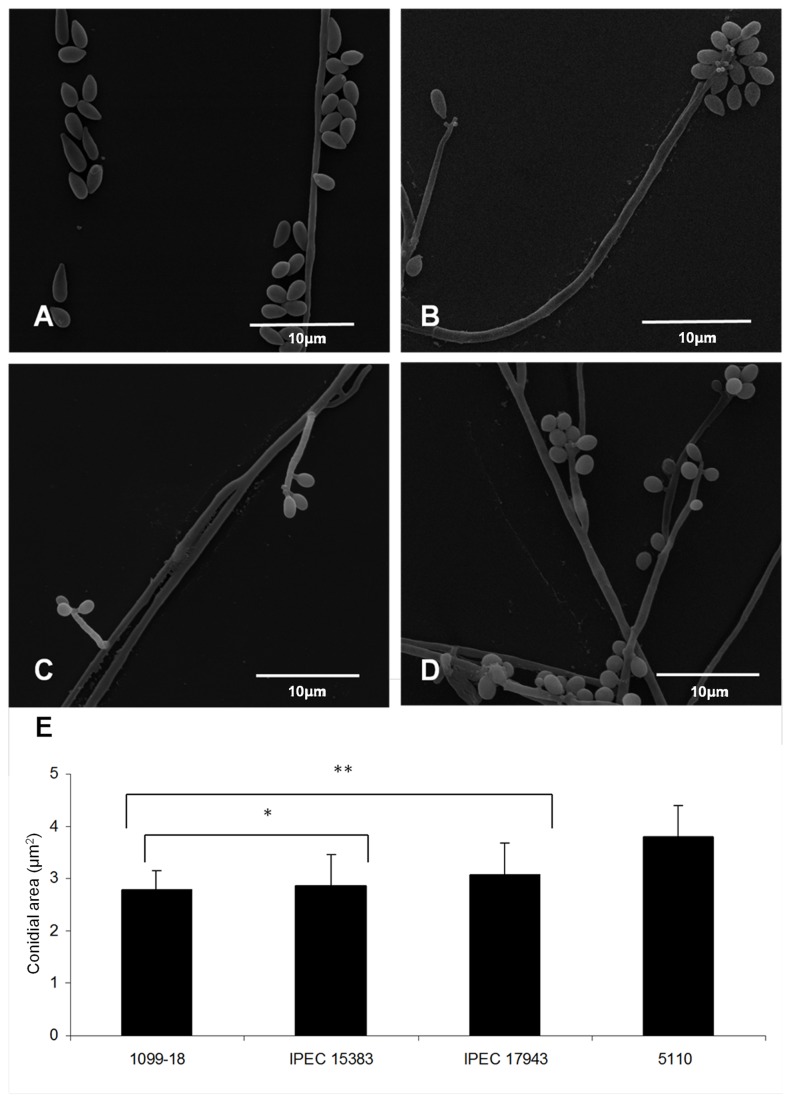
Morphology and morphometry of the mycelial phase of *S. schenckii* and *S. brasiliensis* clinical isolates. (A, B) Scanning electron microscopy of 1099-18 and IPEC 15383 strains of *S. schenckii*, respectively; (C, D) Scanning electron microscopy of IPEC 17943 and 5110 strains of *S. brasiliensis*, respectively. (E) Morphometric analysis of *S. schenckii* and *S. brasiliensis* conidia showing the cell mean size (+/- standard deviation). Forty five conidial cells of each strain were analyzed. *p< 0.05 relative to the *S. brasiliensis* isolates; ** *p*< 0.05 relative to the 5110 isolate.

To compare the morphology between the clinical isolates of *S. schenckii* and *S. brasiliensis*, morphometric analysis was performed using scanning electron microscopy and ImageJ analysis, as detailed in the methodology. The conidia of both *S. schenckii* clinical isolates did not differ in size, presenting an area of 2.78 ± 0.36 and 2.86 ± 0.59 µm^2^ for isolates 1099-18 and IPEC 15383, respectively (Table 3, Figure 2). However, the conidia of *S. brasiliensis* isolates varied statistically in size (*p* < 0.05), featuring an area of 3.07 ± 0.61 and 3.81 ± 0.58 µm^2^ for IPEC 17943 and 5110, respectively (Table 3, Figure 2). Furthermore, *S. schenckii* isolates presented elongated oval conidia, while *S. brasiliensis* isolates showed simply oval conidia (Figure 2). In addition, the 1099-18 isolate (*S. schenckii*) presented predominantly sessile conidia (Figure 2A), while the IPEC 15383 isolate of *S. schenckii* and IPEC 17943 of *S. brasiliensis* showed sympodial conidia (Figure 2B and C). In contrast, the 5110 isolate of *S. brasiliensis* presented both sessile and sympodial conidia (Figure 2D).

**Table 3:** Morphometric characteristics of *S. schenckii* and *S. brasiliensis* conidia.

	***S. schenckii***		***S. brasiliensis***	
**Clinical isolate**	**1099-18**	**IPEC 15383**	**IPEC 17943**	**5110**
**Area + SD (µm^2^)^a^**	**2.78 + 0.36**	**2.86 + 0.59**	**3.07 + 0.61^b^**	**3.81 + 0.58^c^**
**Morphology**	**Elongated oval**	**Elongated oval**	**Oval**	**Oval**
**Disposition of conidia**	**Sessile**	**Sympodial**	**Sympodial**	**Sessile/sympodial**

a SEM morphometric analysis was performed with a mean of 34 conidia per isolate.

b *p*<0.05 compared with *S. schenckii* isolates.

c *p*<0.05 compared with *S. schenckii* isolates and IPEC 17943.

**Figure 3:**
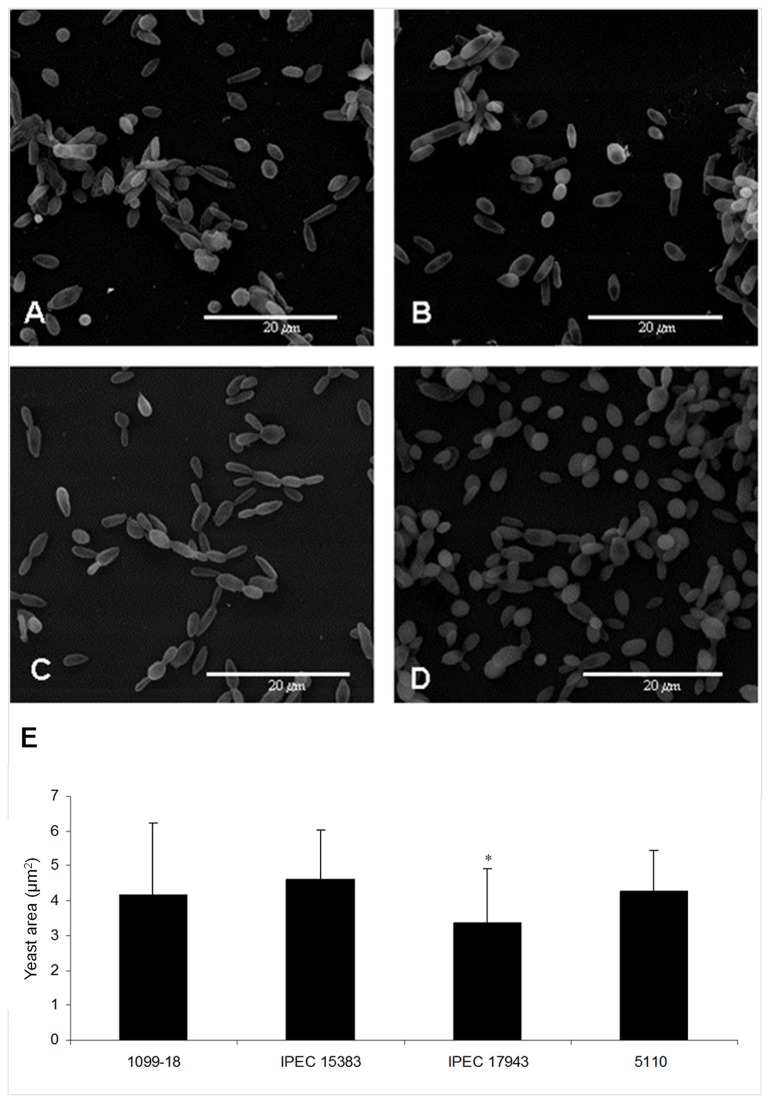
Morphology and morphometry of the yeast phase of *S. schenckii* and *S. brasiliensis* clinical isolates. (A, B) Scanning electron microscopy of 1099-18 and IPEC 15383 of *S. schenckii*, respectively; (C, D) IPEC 17943 and (D) 5110, *S. brasiliensis* Scanning electron microscopy of IPEC 17943 and 5110 strains of *S. brasiliensis*, respectively. (E) Morphometric analysis of yeast cells of *S. schenckii* and *S. brasiliensis* isolates showing the cell mean size (+ standard deviation). Fifty eight yeast cells of each strain were analyzed. * *p*< 0.05 relative to all other isolates.

Yeast cell morphometry revealed no differences in size among 1099-18, IPEC 15383 (*S. schenckii*) and 5110 (*S. brasiliensis*) isolates; however, the yeast cells of the less virulent *S. brasiliensis* isolate, IPEC 17943, were significantly smaller (Table 4, Figure 3). Moreover, image analysis revealed a pleomorphism of the yeast-like phase, which varied from oval to elongated (cigar-shaped), independent of the species and isolate origin (Figure 3). For the *S. brasiliensis* IPEC 17943 isolate, a predominance of elongated yeast cells was observed (Figure 3C), while the 5110 isolate contained oval yeast cells (Figure 3D).

**Table 4:** Morphometric characteristics of yeast cells of *S. schenckii* and *S. brasiliensis* clinical isolates.

	***S. schenckii***		***S. brasiliensis***	
**Clinical isolate**	**1099-18**	**IPEC 15383**	**IPEC 17943**	**5110**
**Area + SD (µm^2^)^a^**	**4.18 + 2.03**	**4.60 + 1.44**	**3.38 + 1.53^b^**	**4.28 + 1.16**
**Morphology**	**elongated**	**elongated**	**elongated**	**oval**

a SEM morphometric analysis was performed with a mean of 58 yeast cells per isolate

b *p*<0.05, compared with all other isolates.

### Cell wall topography and gp70 expression

**Figure 4:**
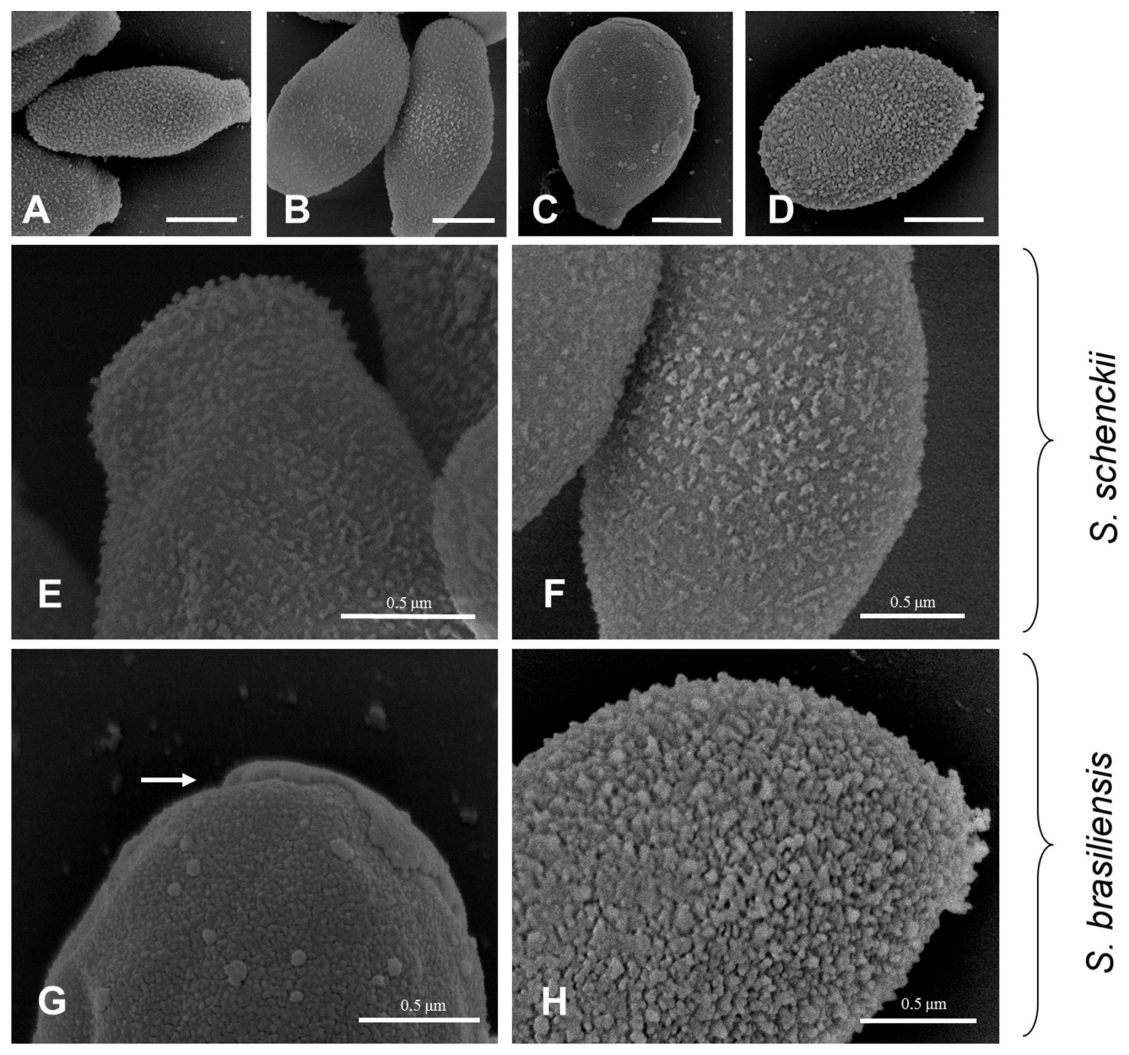
Scanning electron microscopy (SEM) of the cell surface of *S. schenckii* and *S. brasiliensis* yeast cells. (A, E) 1099-18; (B, F) IPEC 15383; (C, G) IPEC 17943 and (D, H) 5110. For the 1099-18, IPEC 15383 and 5110 strains the outer cell layer shows a dense amorphous fibrillar material. The white arrow indicates a cell wall fracture observed in the IPEC 17943 strain. (A-D) Scale bar -1.0 µm.

High resolution scanning electron microscopy images demonstrated a similar topography of the yeast cell surface for both *S. schenckii* isolates (Figure 4, panels A/B and E/F). In addition, a smooth irregular cell surface indicating the presence of an abundant amorphous material was observed in the *S. brasiliensis* 5110 isolate (Figure 4D and H). Nevertheless, this isolate has a denser surface compared with the *S. schenckii* isolates. In contrast, the *S. brasiliensis* IPEC 17943 isolate presents a compact yeast cell surface (Figure 4C and G). In this case, some cells showed fractured outer cell wall areas, as illustrated in Figure 4G.

**Figure 5:**
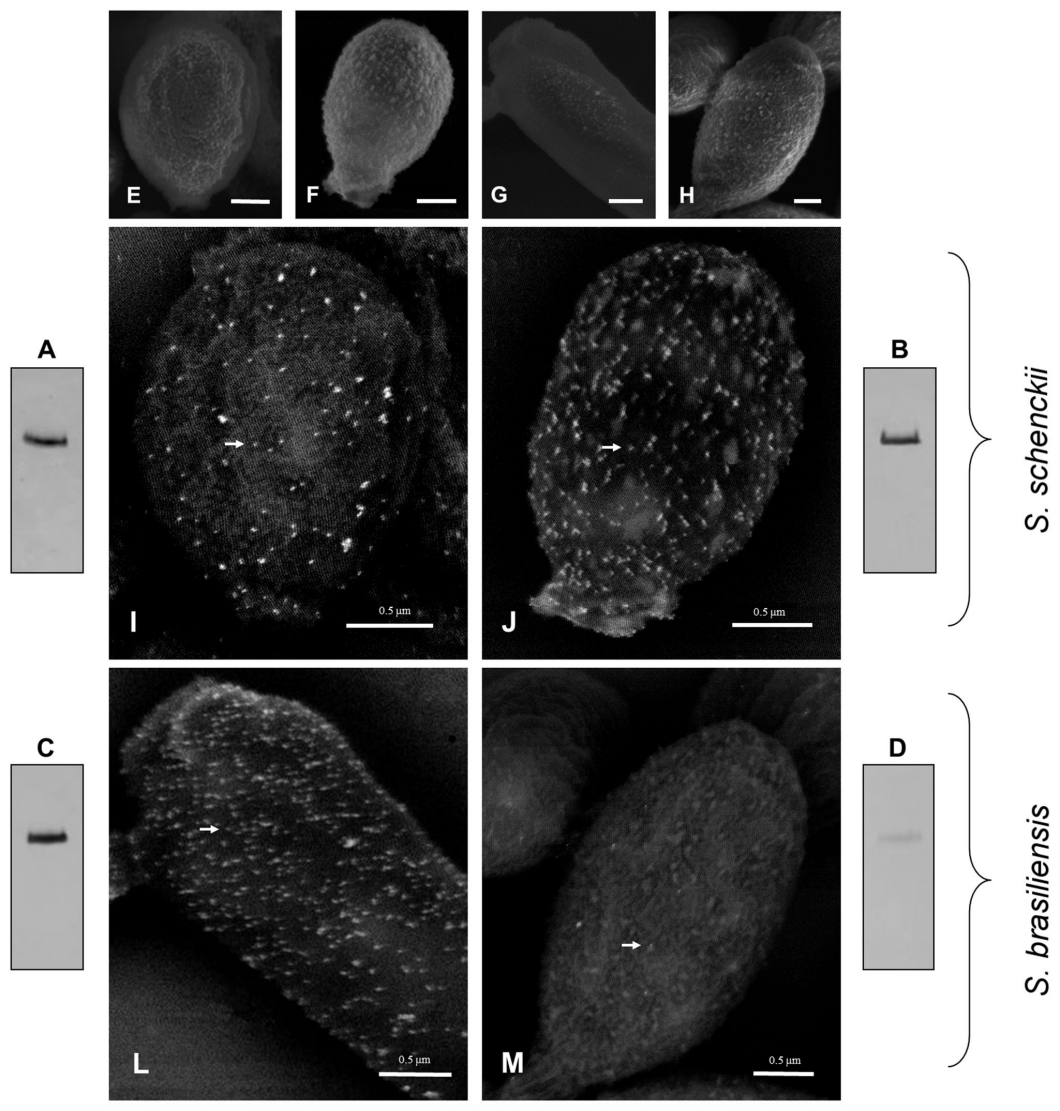
Gp70 expression on the cell wall of *S. schenckii* and *S. brasiliensis* clinical isolates. The expression of gp70 in cell wall extracts (see Methods) was verified by western blot analysis of *S. schenckii* 1099-18 (A) and IPEC 15383 (B); and (C) of *S. brasiliensis* IPEC 17943 (C) and 5110 (D). The amount of protein loaded was 5 µg (A-C) and 15µg (D). (E-M) Scanning electron microscopy showing the backscattered electron imaging (I-M) of yeast cells of *S. schenckii* 1099-18 (E, I), IPEC 15383 (F, J) and of *S. brasiliensis* IPEC 17943 (G, L) and 5110 (H, M) which were incubated with a monoclonal antibody anti-gp70 followed by a mouse anti-IgG gold-conjugate. E-H, Scale bar 1.0 µm.

In parallel, the yeast cell surface of *S. schenckii* and *S. brasiliensis* were analyzed by field emission scanning electron microscopy. Yeast cells of all four isolates were incubated with the anti-gp70 monoclonal antibody followed by labeling with a 15-nm colloidal gold-conjugated anti-mouse IgG. A backscattered electron detector was used to reveal the sites where the gold particles were attached, showing a positive labeling on the cell surface of both *S. schenckii* isolates (Figure 5E-M). Interestingly, a higher density of antibody binding sites was also observed for the yeast cells of *S. brasiliensis* IPEC 17943 isolate (Figure 5L). In contrast, poor labeling was observed on the yeast cell surface of the *S. brasiliensis* 5110 isolate (Figure 5M). In a western blot assay, a weakly positive band recognized by the anti-gp70 mAb P6E7 was observed on the 5110 cell surface extract only when a three-fold protein concentration was loaded on the 1D gel (Figure 5D) compared with the other isolates (Figure 5A-C). These observations corroborate the SEM results.

**Figure 6:**
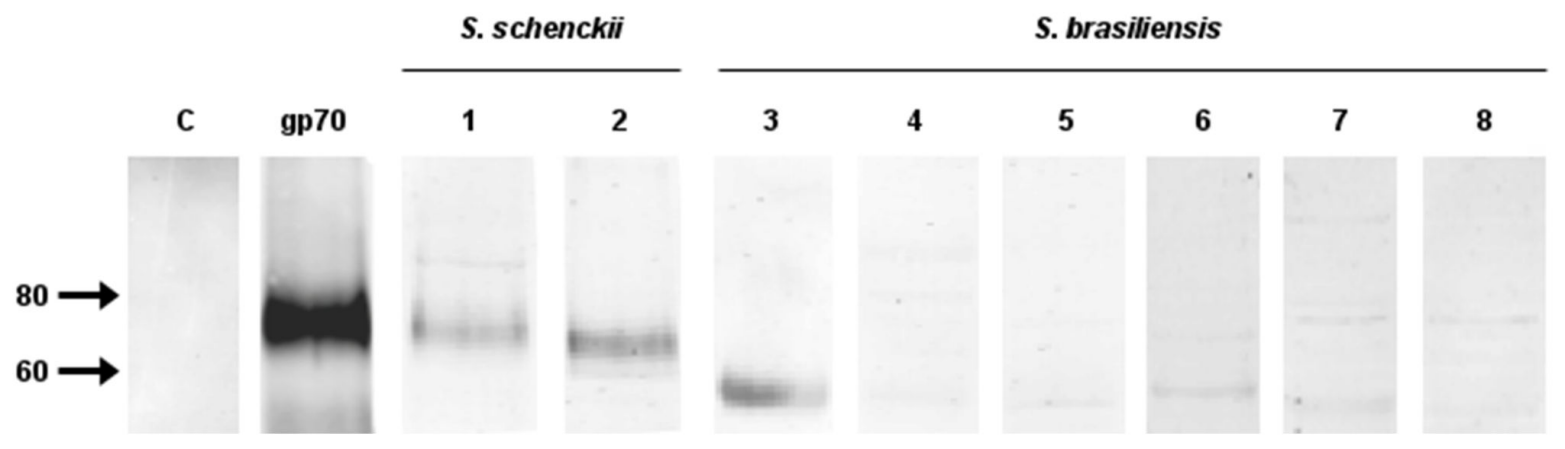
Western blot analysis of the cell surface gp70 of *S. brasiliensis*. The gp70 antigen was revealed on cell surface extracts of the yeast parasitic phase of two *S. schenckii* reference strains (lanes 1 and 2) and several clinical isolates of *S. brasiliensis* (lanes 3 to 8) by a monoclonal antibody anti-gp70, mAb P6E7. A purified gp70 was used as a positive control and in C is shown the negative control of the mAb P6E7. Correspondent strains in lanes (1) 1099-18; (2) IPEC 15383; (3) IPEC 17943; (4) 5110; (5) Ss 54; (6) UFTM 01; (7) HUPE 114500 and (8) HUPE 114158. The amount of protein loaded in lanes 1 to 8 was 5 µg..

Once differences in gp70 expression were observed among *S. brasiliensis* isolates showing divergent virulence profiles, the presence of gp70 in cell wall extracts of all *S. brasiliensis* isolates was comparatively analyzed by western blot using a similar protein load (Figure 6). As expected, for *S. schenckii* reference strains, a positive band with an apparent MW in the range of 60-70 kDa was detected. Interestingly, the *S. brasiliensis* clinical isolates exhibited almost no reactive band, excepting the IPEC 17943 strain (Figure 6, lane 3).

### Subcellular distribution of gp70 in *S. schenckii* and *S. brasiliensis*

**Figure 7:**
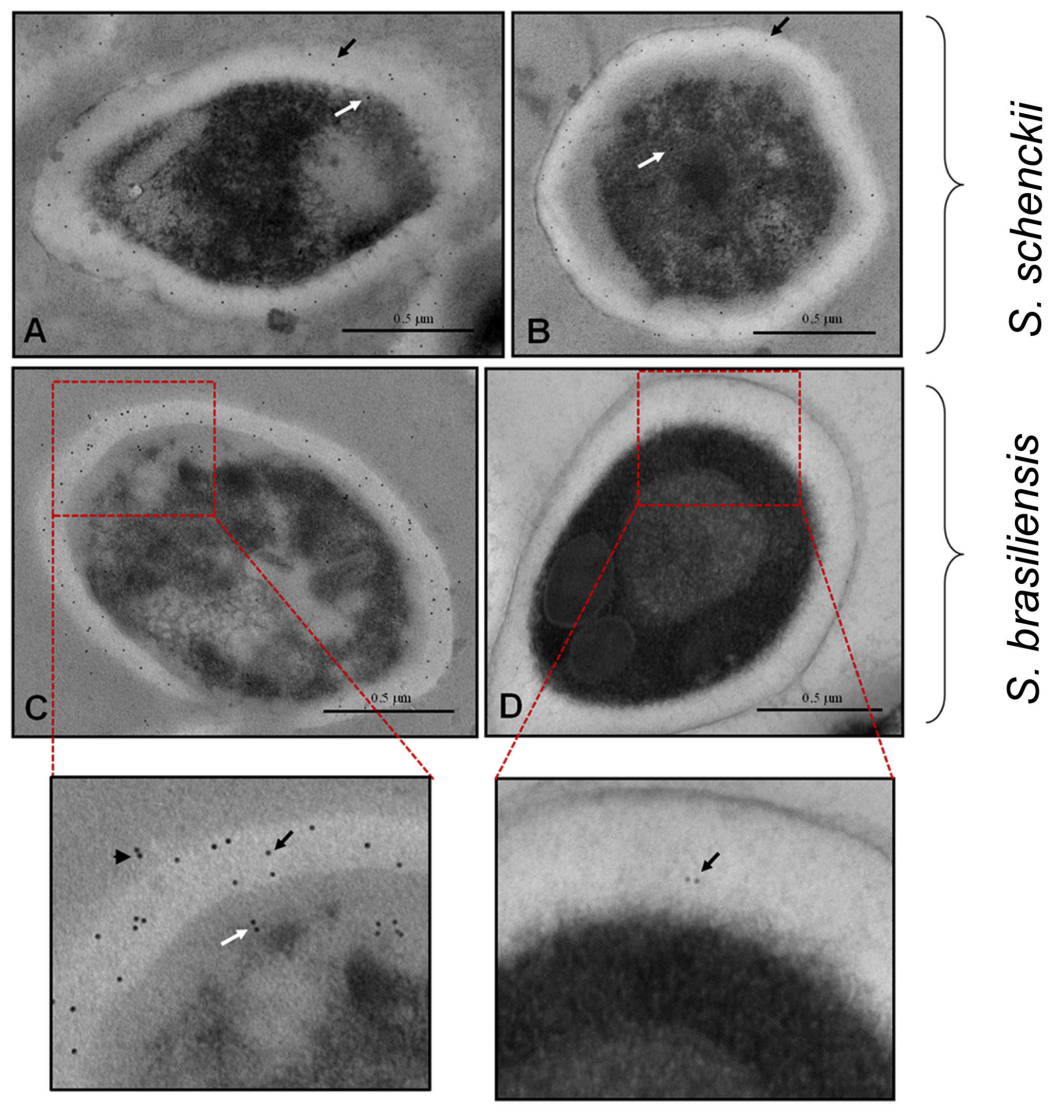
Distribution and subcellular localization of gp70 on yeast cells of *Sporothrix* clinical isolates. Transmission electron micrograph of *S. schenckii* or *S. brasiliensis* yeast cells incubated with the monoclonal anti-gp 70 antibody followed by a mouse anti-IgG gold-conjugated antibody. Is shown in (A) *S. schenckii* 1099-18, (B) *S. schenckii* IPEC 15383, (C) *S. brasiliensis* IPEC 17943 and (D) *S. brasiliensis* 5110. Arrows indicate localization of gp70 on the cell wall (black arrows), in the cytoplasm (white arrows) and at the extracellular compartment (arrowheads). Images featured were digitally magnified.

The subcellular distribution of gp70 in *S. schenckii* and *S. brasiliensis* yeast cells was investigated using transmission electron microscopy. Images of yeast sections incubated with mAb P6E7 and a 10-nm colloidal gold-conjugated anti-mouse IgG revealed higher positive labeling on the cell wall of IPEC 17943 *S. brasiliensis* yeast cells (Figure 7C) and in both *S. schenckii* strains (Figure 7A and B). In addition, the images demonstrated the low labeling of the cell wall of the *S. brasiliensis* 5110 isolate (Figure 7D), which corroborates the scanning electron microscopy and western blot data.

Furthermore, the presence of gold particles in the intracellular space suggests that gp70 is distributed in several subcellular compartments and is not restricted to the cell surface (Figure 7). Interestingly, a positive labeling of gp70 in the extracellular space was observed (Figure 7C, amplified area), suggesting that gp70 can also be secreted.

### Identification and *in silico* characterization of the cell surface gp70

The identification of the gp70 cell wall antigen was performed for both *S. schenckii* and *S. brasiliensis* using a 1D-MALDI/TOF/TOF approach. From the cell wall extracts, the corresponding bands that were recognized by the monoclonal antibody anti-gp70 were excised from SDS-PAGE gels and tryptic digested for MS/MS identification and sequencing (Table 5). A 2D gel was also run to ascertain that the identified peptides corresponded to a single component, gp70 (data not shown). In addition, a purified gp70 antigen [28] was also sequenced to confirm the peptide sequences (Table 5).

**Table 5:** Identified peptide sequences of gp70 by MS/MS.

***Sporothrix* spp**	**ORF**	**Peptide**
*S. schenckii*	Ss06314	AVYVTSNTEHNSVVAIPIAR
		GPTNTVSHVFFSGDQETVFTTVK
		TVIPGQDATCWVAICPATHTAFVTDIR
		KPVQHALLTPLGLDR
*S. brasiliensis*	Sb05019	AVYVTSNTEHNSVVAIPIAR
		GPTNTVSHVFFSGDQETVFTTVK
		TVIPGQDATCWVAICPATHTAFVTDIR
		LVEMSLANAEIIGEPIDLTTFSNDPGLTEIR
		KPVQHALLTPLGLDR

**Figure 8:**
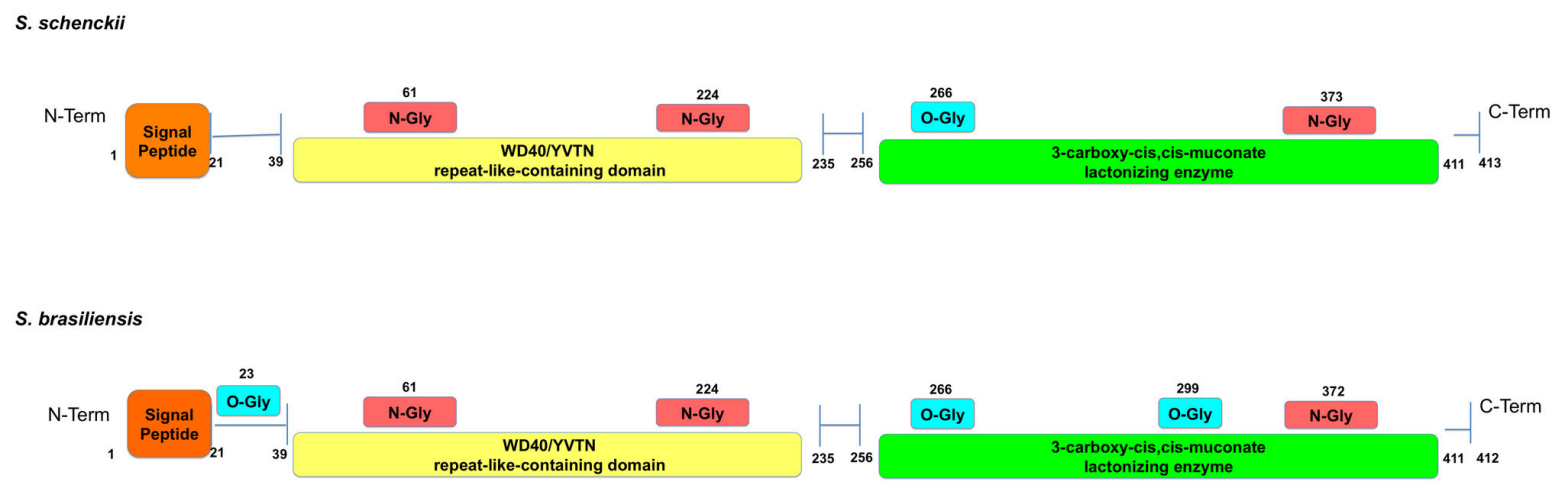
*In silico* characterization of gp70 in *S. schenckii* (1099-18) and *S. brasiliensis* (5110). Identification of important domains and putative glycosylation sitesin the gp70 protein sequence using InterPro and EnsembleGly. The gene and protein sequences of gp70 were deposited in the GenBank and EMBL-EBI, respectively.

The identified peptide sequences shown in Table 5 were deposited at EMBL-EBI as corresponding to cell wall gp70 for both *Sporothrix* species. The identification was based on the genome databases of *S. schenckii* and *S. brasiliensis* (unpublished results), and the sequences correspond to the ORFs Ss06314 and Sb05019, respectively. Interestingly, these sequences present a 3-carboxymuconate cyclase domain (Figure 8), an enzyme involved in benzoate degradation. The *gp70* gene encodes a 1,242bp and 1,239bp ORF interrupted by a 64bp intron in *S. schenckii* (Genbank accession number KF275147) and *S. brasiliensis* (Genbank accession number KF275146), respectively.

According to InterPro analysis, these genes contain a signal peptide (1-21 aa), a WD40/YVTN repeat-like-containing domain (39-235 aa) and a 3-carboxy-cis,cis-muconate lactonizing enzyme domain (256-411 aa) (Figure 8). Analysis by the SignalIP 4.1 predictor shows the presence of a canonical secretion signal, while an analysis using the BIG-PI Fungal predictor indicates the lack of a GPI anchor (data not shown). Furthermore, gp70 was investigated for the presence in its sequence of potential glycosylation and phosphorylation sites using the EnsembleGly and NetPhos software, respectively. Interesting differences were observed, as the gp70 of *S. brasiliensis* shows three putative O-glycosylation sites while only one was predicted for the *S. schenckii* glycoprotein. The number of predicted N-glycosylation sites is similar with slight differences in the sequences inferred by the EnsembleGly algorithm (Figure 8). NetPhos analysis suggested that gp70 of both species has 20 putative phosphorylation sites.

## Discussion

Until recently, sporotrichosis was known as a benign subcutaneous mycosis caused by a single etiological agent, *S. schenckii* stricto sensu. This clinical and epidemiological panel has changed dramatically with the emergence of *S. brasiliensis*, a cryptic species of the *S. schenckii* complex, which is predominant in Brazil [10,15]. *S. brasiliensis* is related not only to the zoonotic transmission of this disease but also to the emergence of severe cases of sporotrichosis [37-39]. One question that arises is whether *S. schenckii* and *S. brasiliensis* share similar virulence factors and/or have the same pathogenicity. To attempt to answer this question, at least in part, several clinical isolates of *S. brasiliensis* were analyzed in the present work for their virulence capacity in a subcutaneous murine model compared with two *S. schenckii* reference strains. Six clinical isolates of *S. brasiliensis* were used in this study, two of which have been previously described [14,38]. For all other clinical isolates, a phylogenetic distribution was established to group them into the corresponding cryptic species inside the *Sporothrix* spp. (Figure S1). Four strains were further deposited in the American Type Culture Collection (ATCC) with the following reference numbers: ATCC MYA-4820 (*S. schenckii* IPEC 15383), ATCC MYA-4821 (*S. schenckii* 1099-18), ATCC MYA-4823 (*S. brasiliensis* 5110) and ATCC MYA-4824 (*S. brasiliensis* IPEC 17943). A morphometric study was also performed with either conidia or yeast cells of these four strains. Interestingly, the conidial morphology of these organism supports differences found in the phylogenetic distribution because *S. brasiliensis* presented only oval conidia and *S. schenckii* isolates presented both elongated and oval conidia.

A subcutaneous murine model of infection was established to compare the virulence and dissemination capacity of the six *S. brasiliensis* clinical isolates that were originally associated with distinct hosts and/or clinical manifestations of sporotrichosis. The results show that, with one exception (*S. brasiliensis* IPEC17943), all other clinical isolates of *S. brasiliensis* had increased pathogenicity compared with *S. schenckii*. Furthermore, several parameters that could be related to the differences observed in the virulence of *S. brasiliensis* were investigated.

In a previous study, it was shown that several antigens secreted by *S. schenckii istricto* sensu were able to induce a specific humoral response in infected animals, specially the antigen of 70 kDa (gp70), indicating a plausible participation of specific antibodies against this molecule in the control of infection [28,40]. Nascimento and co-workers had studied the M64 strain (*S. schenckii*, ATCC MYA-4822), which was unable to kill infected mice in a murine model of disseminated sporotrichosis [28]. Altogether, the observations indicate that a protective immune response during infection can be associated with the cell wall gp70-expressing isolates, although the outcome of the infection cannot be limited to a single antigen or adhesin [24].

The importance of this glycoprotein during infection was demonstrated by the treatment of mice infected with *S. schenckii* with a monoclonal antibody against gp70. The results showed a significant reduction in the number of CFUs in the organs of mice when the mAb was injected before and during *S. schenckii* infection [28]. A recent study demonstrated that the fungicidal ability and TNF-α production of macrophages increased when the fungus was phagocytized in the presence of immune-inactivated serum or mAb P6E7 [41]. It is tempting to speculate that anti-gp70-mediated phagocytosis may be essential to macrophage killing, consequently producing TNF-α to control the sporotrichosis. This molecule also acts as an adhesin and interacts with fibronectin, an extracellular matrix protein [24]. These observations indicate that gp70 can play a dual role in the host-fungus interplay and could be compared, in its function, to a well described immunodominant antigen of the dimorphic fungus *Blastomyces dermatitidis*, BAD-1. BAD-1 protein which is a secreted and cell surface-localized virulence factor play a role as both an adhesin and immunomodulatory molecule [42,43].

Our hypothesis was that the cell surface gp70 antigen could have a direct association with the virulence profile of the pathogenic species within the *S. schenckii* complex. No previous reports had proven the presence or expression of gp70 in *S. brasiliensis*. In the present study, gp70 was immune-localized on the surface of two clinical isolates of *S. brasiliensis* that were selected because they exhibit lower and higher virulence capacity in a subcutaneous murine model. The results show that a greater expression of gp70 on the fungal surface correlates with a lower virulence profile of the strain. In addition, a western blot approach was applied to relatively quantify the differences in the gp70 cell wall expression of all *S. brasiliensis* clinical isolates and its relation to the severity of the infection. A clear correlation was observed, as a reduced level of gp70 expression was found in all virulent *S. brasiliensis* isolates. This finding could represent a misbalance in the immune response, as this antigen induces a protective host response [44]. Accordingly, the *S. schenckii* clinical reference strains of different geographical origins (IPEC 15383 and 1099-18), which were both less virulent in the subcutaneous murine model, presented high expression of gp70. No differences in the cell surface topography or cell morphometry were observed in *S. schenckii*. The low virulence isolate of *S. brasiliensis*, IPEC17943, parallels the virulence profile and gp70 expression of *S. schenckii*.

The identities of the gp70 antigen of *S. schenckii* and *S. brasiliensis* were further investigated using proteomics. Previously, Nascimento and co-workers [28] suggested a possible amino acid sequence for the gp70 exoantigen based on a peptide mass fingerprint and MS/MS data without any available *S. schenckii* genomic database, which could be used to perform a confident protein identification. In the present work, the genome database of *S. schenckii* and *S. brasiliensis* (unpublished results) was used to perform an accurate identification of gp70 using MALDI-ToF/MS. The identification of gp70 on cell wall extracts of either *S. schenckii* or *S. brasiliensis* allowed us to align the protein sequences and observe differences in their primary structure. The *gp70* gene encodes a carboxy-cis,cis-muconate cyclase (EC 5.5.1.5) present in the β-ketoadipate pathway, as illustrated in Figure S5. In filamentous fungi, this enzyme plays a key role in the metabolism of aromatic compounds to produce acetyl-CoA, which is processed by the TCA cycle [45]. Surprisingly, in the tomato wilt pathogen *Fusarium oxysporum* f. sp. *lycopersici*, mutants of carboxy-cis,cis-muconate cyclase are impaired in root invasion and are nonpathogenic. The authors hypothesize that the β-ketoadipate pathway in plant-pathogenic, soil-borne fungi is necessary to degrade phenolic compounds in roots to establish disease [46]. In the case of human pathogens, the function of this enzyme remains unknown. The evidence shown here that this glycoprotein is localized to several subcellular locations, including the cell surface, may suggest that gp70 can play a dual role depending on its cellular localization in the fungal cell. This would not be the first time that a secreted and cell associated protein would be involved in adhesion and immunomodulation of host response, as mentioned before [42].

Furthermore, differences in the putative glycosylation sites of gp70 were observed when comparing the entire sequences of this glycoprotein from *S. schenckii* and *S. brasiliensis*. No evidence of a GPI anchor was found according to the predictor BigPI. Interestingly, the protein has a predicted secretion signal. It is plausible to infer that the differences in fungal virulence may also be related to the presence of gp70 isoforms or glycoforms rather than being limited to its expression levels. Other cell surface differences were observed between *S. schenckii* and *S. brasiliensis*, as shown by SEM and MET, and will need further investigation.

Here, for the first time, the identification, expression level and subcellular localization of gp70 of *S. brasiliensis* and *S. schenckii* and its possible correlation with the virulence profile of these pathogenic species are reported.

## Supporting Information

Figure S1 **Phylogenetic distribution of *Sporothrix* spp. isolates through used for cell surface analysis and gp70 expression.** Phylogenetic trees for cal locus was inferred using the Maximum Likelihood and Nighbor-Joining methods. Bootstrap values representing the branch fidelity were inferred from 1000 replicates and added in the tree partitions. Sequences representing type strains of *S. schenckii*, *S.* brasiliensis and *S. globosa* were added for genotyping. Isolates analyzed in this work are tagged with if clustered in *S. brasiliensis* species or ● for *S. schenckii* clade.
(TIF)

Figure S2 **Scheme of the subcutaneous murine model of sporotrichosis.** The mycelium phase of *Sporothrix* spp was cultivated in Sabouraud broth at 25°C. Conidia was isolated by filtration in sterilized gauze and the cell number counted in a Neubauer chamber. Thereafter, BALB/C male mice were trichotomized in dorsal sacral region to undergo the subcutaneous inoculation, as illustrated.
(TIF)

Figure S3 **Macroscopic aspect of the cutaneous lesions caused by *Sporothrix schenckii* and *Sporothrix brasiliensis*.** Mice were inoculated with *Sporothrix* sp. conidia and the progress of sporotrichosis was observed during 40 days, at least. The aspect of the primary lesion of mice infected with each strain listed in Table 1 is shown at day 40^th^ post-infection.
(TIF)

Figure S4 **Histopahology showing the phagocytic mononuclear reaction and the parasite burden in skin specimens.** (A, C and E) Hematoxylin and eosin stain and (B, D and F) Grocott-gomori stain of skin specimens of mice infected with (A, B) *S. brasiliensis* (strain IPEC 17493) showing a poorly formed granuloma (black arrow); (C, D) *S. brasiliensis* (strain 5110) showing a mononuclear infiltrate without granuloma and a high fungal load in the tissue and, (E, F) *S. schenckii* (strain 1099-18) showing a poorly formed granuloma (black arrow) and few yeast cells.
(TIF)

Figure S5 **Characterization of gp70 by a genomic approach.** (A) Chemical reaction catalyzed by the carboxy-cis,cis-muconate cyclase enzyme; (B) Distribution of carboxy-cis,cis-muconate cyclase using Maximum Likelihood inferences among Sordariomycete fungi.
(TIF)
