# Supplementary figures and images for: Differences in Cell Morphometry, Cell Wall Topography and Gp70 Expression Correlate with the Virulence of Sporothrix brasiliensis Clinical Isolates
Source: PLoS One. 2013 Oct 7;8(10):e75656. doi: 10.1371/journal.pone.0075656 (PMC3792129; doi:10.1371/journal.pone.0075656)

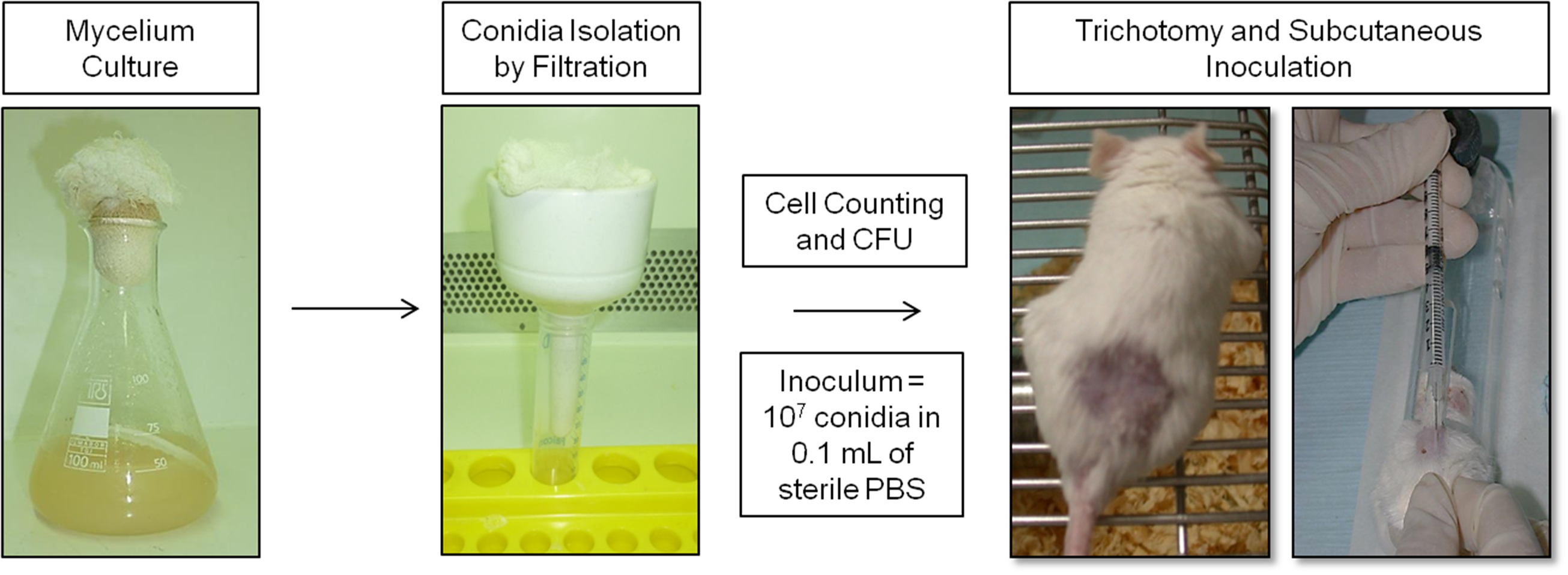

Supplement: Figure S2 — Scheme of the subcutaneous murine model of sporotrichosis. The mycelium phase of Sporothrix spp was cultivated in Sabouraud broth at 25°C. Conidia was isolated by filtration in sterilized gauze and the cell number counted in a Neubauer chamber. Thereafter, BALB/C male mice were trichotomized in dorsal sacral region to undergo the subcutaneous inoculation, as illustrated. (TIF) [file pone.0075656.s002.tif]

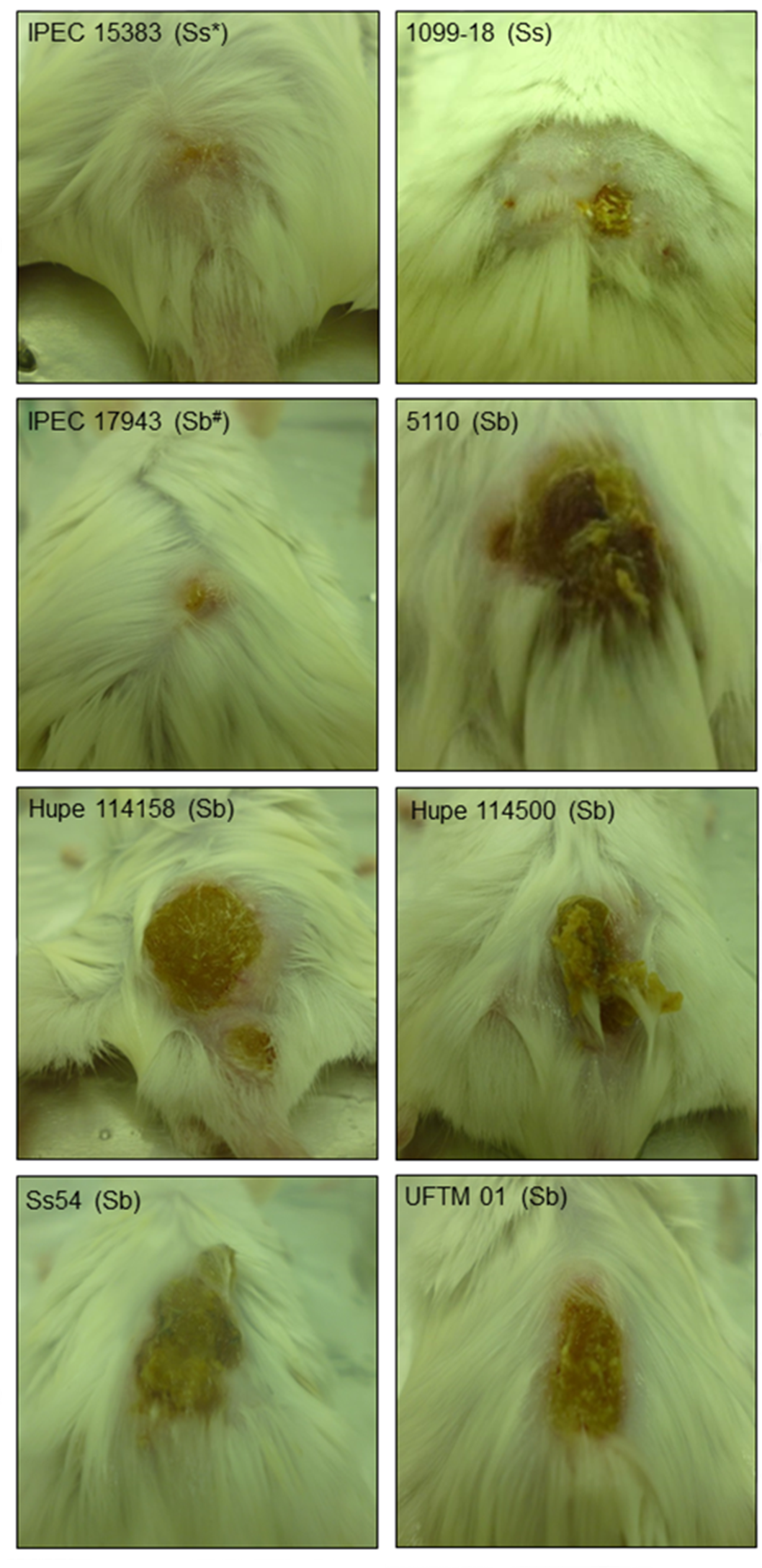

Supplement: Figure S3 — Macroscopic aspect of the cutaneous lesions caused by Sporothrix schenckii and Sporothrix brasiliensis. Mice were inoculated with Sporothrix sp. conidia and the progress of sporotrichosis was observed during 40 days, at least. The aspect of the primary lesion of mice infected with each strain listed in Table 1 is shown at day 40th post-infection. (TIF) [file pone.0075656.s003.tif]

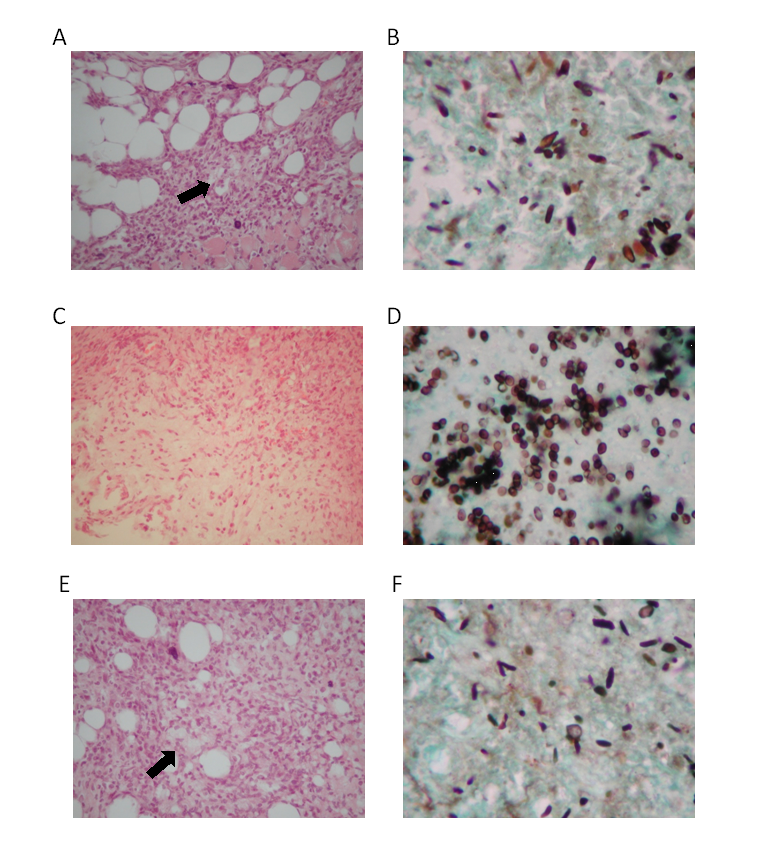

Supplement: Figure S4 — Histopahology showing the phagocytic mononuclear reaction and the parasite burden in skin specimens. (A, C and E) Hematoxylin and eosin stain and (B, D and F) Grocott-gomori stain of skin specimens of mice infected with (A, B) S. brasiliensis (strain IPEC 17493) showing a poorly formed granuloma (black arrow); (C, D) S. brasiliensis (strain 5110) showing a mononuclear infiltrate without granuloma and a high fungal load in the tissue and, (E, F) S. schenckii (strain 1099-18) showing a poorly formed granuloma (black arrow) and few yeast cells. (TIF) [file pone.0075656.s004.tif]

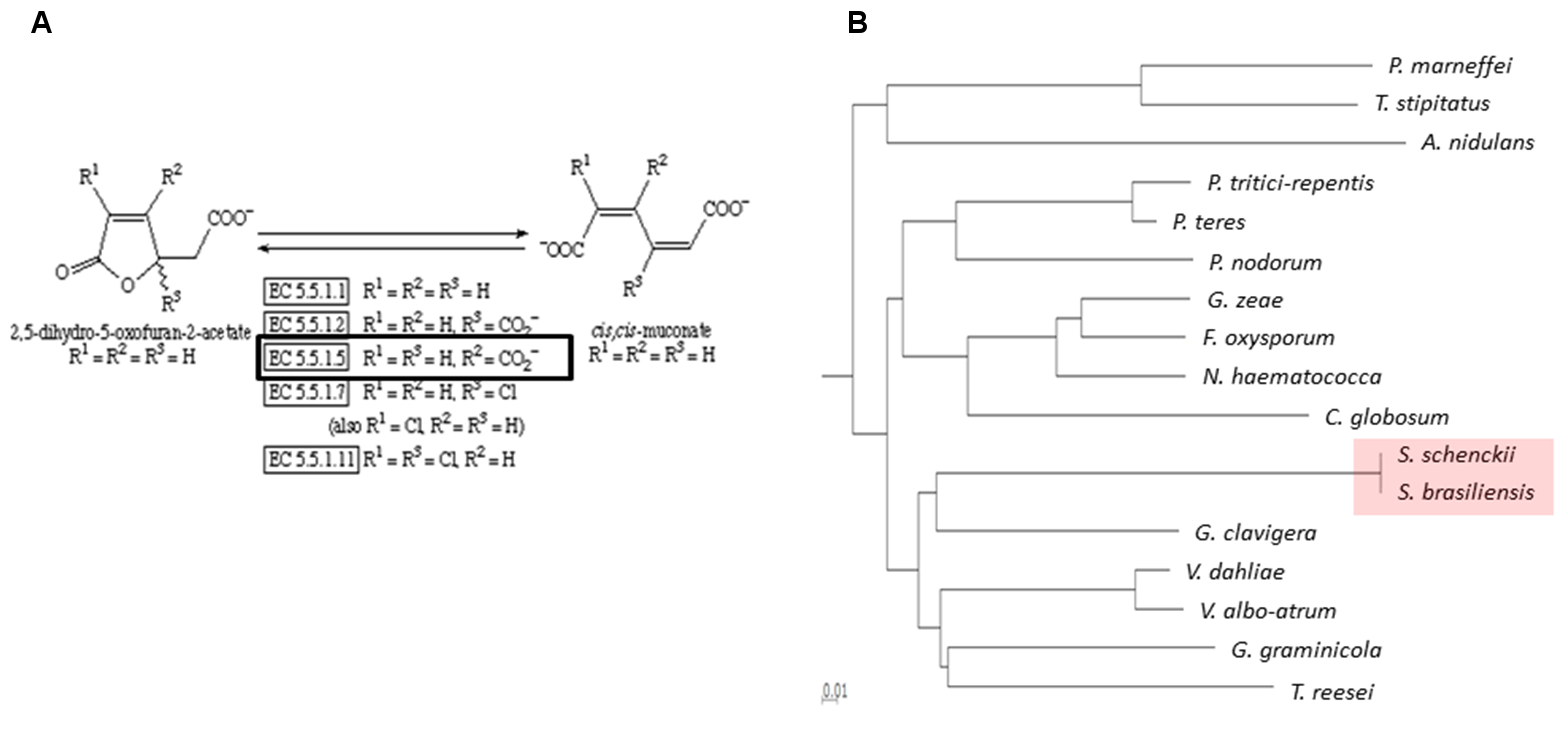

Supplement: Figure S5 — Characterization of gp70 by a genomic approach. (A) Chemical reaction catalyzed by the carboxy-cis,cis-muconate cyclase enzyme; (B) Distribution of carboxy-cis,cis-muconate cyclase using Maximum Likelihood inferences among Sordariomycete fungi. (TIF) [file pone.0075656.s005.tif]
